# Histological and immunohistochemical analysis of human periapical lesions: a study of TGF-β1 and CD68 markers

**DOI:** 10.1186/s12903-025-05845-2

**Published:** 2025-04-11

**Authors:** Nermeen AbuBakr, Geraldine M. Ahmed, Amany Hany Mohamed Kamel

**Affiliations:** 1 Oral Biology Department, Faculty of Dentistry, Cairo University, Cairo, Egypt; 2 Department of Endodontics, Faculty of Dentistry, Cairo University, Cairo, Egypt

**Keywords:** Radicular cyst, Periapical granuloma, Immunohistochemistry, Inflammation, Macrophages

## Abstract

**Background:**

Various inflammatory and anti-inflammatory mediators, along with diverse cell types, are implicated in the development and progression of periapical lesions. This work aimed to assess the immuno-expression of transforming growth factor-beta 1 (TGF-β1) and CD68 (a macrophage marker), elucidating their roles and potential correlations. Additionally, histological analysis was conducted to evaluate the intensity of inflammatory infiltrates in chronic periapical lesion samples.

**Methods:**

Tissue samples from fifty individuals with chronic periapical lesions [25 radicular cysts (RCs) and 25 periapical granulomas (PGs)] were obtained, along with control samples from four healthy third molars’ dental pulp. Histological examination and inflammatory infiltrate categorization were performed. Immunohistochemical analysis of TGF-β1 and CD68 markers, along with morphometric assessment, were conducted.

**Results:**

The control group displayed normal, inflammation-free pulp tissues, while intense inflammation was observed in PGs and RCs (Score 4 and 3, respectively) dominated by macrophages, plasma cells, and lymphocytes. Immunohistochemistry showed higher TGF-β1 and CD68 expression in PGs and RCs versus control (*P* < 0.001). Moreover, PGs exhibited greater TGF-β1 and CD68 expression than RCs (*P* < 0.001). However, a negative relationship was detected between the 2 markers (*P* < 0.05).

**Conclusions:**

This study highlighted varying expressions of TGF-β1 and CD68 in PGs and RCs, indicating their potential roles in lesion pathology. However, a negative correlation between these markers was observed. Accordingly, their precise role in periapical lesion progression and repair requires further investigation.

## Introduction

Periapical lesions, specifically apical periodontitis (AP), represent the most common pathological conditions associated with untreated or inadequately managed endodontic treatments [1]. The development of chronic root canal infections triggers a complex inflammatory response that results from both microbial infection and an immunological defense reaction aimed at safeguarding against the spread of infectious agents [2, 3].

Histological analysis has long served as the benchmark, enabling the definitive characterization of the clinical manifestations of AP from lesions of alternative origins [4]. Periapical lesions are commonly categorized based on their morphology, radiographic extent, and clinical presentations, primarily grouping into three distinct categories: periapical abscesses, periapical granulomas (PGs), and radicular cysts (RCs) [5]. Periapical abscesses are characterized by pus formation due to altered cellular processes triggered by an acute infection, whereas PGs comprise granulation tissue containing inflammatory cells and fibroblasts. A widely discussed hypothesis suggests that PGs may transform into RCs through stimulation of Malassez epithelial remnants by the body’s immune response [3, 6].

Despite significant progress in understanding the pathogenesis of periapical lesions, notable gaps remain regarding the molecular mechanisms that drive their formation and progression [7, 8]. The inflammatory response within root canals involves a highly coordinated signaling cascade that integrates the immune system’s reactions with microbial activity within the infected tissues [9, 10].

Transforming growth factor-beta 1 (TGF-β1) is a multifunctional cytokine secreted by numerous cell types, including platelets, fibroblasts, macrophages, and inflammatory cells. It plays a vital role in a wide range of cellular and immune functions, including regulation of inflammatory responses [11, 12]. Known for its dual immunomodulatory roles, TGF-β1 can act as both a pro-inflammatory and anti-inflammatory mediator, depending on the signaling pathways it activates [13]. It has been implicated in periapical disease pathology [14, 15]; however, its precise role in lesion development remains poorly understood [8, 10]. Previous studies on TGF-β1 expression in periapical lesions have reported inconsistent findings, particularly regarding its levels in PGs and RCs, underscoring the need for further research [10, 16, 17].

Macrophages are central to orchestrating immune responses in periapical lesions, producing inflammatory mediators when encountering bacterial infections in root canals [18]. They play a critical role in both initiating and sustaining chronic inflammatory processes and serve as a first line of defense in combating microbial invasion [18, 19]. Additionally, macrophages secrete matrix metalloproteinases, which modulate inflammatory processes by activating pro-inflammatory and immunoregulatory mediators, including TGF-β1 [20].

CD68, a widely used marker for macrophages, is essential for understanding the extent and nature of macrophage-mediated inflammation in periapical lesions [21]. Although the role of CD68 has been studied in various oral conditions, its differential expression in PGs and RCs, as well as its correlation with other inflammatory mediators such as TGF-β1, remains underexplored [8].

Thus, this study aimed to investigate the immuno-expression of TGF-β1 and CD68 in PGs and RCs and to examine their correlation—an aspect previously unexplored. Comparative data on the immuno-expression of these key inflammatory mediators remain limited, and the specific interactions between these markers and their roles in disease progression are not fully understood. Understanding how these markers shape the distinct inflammatory microenvironments of these lesions is essential for elucidating their contributions to disease progression and transformation.

## Materials and methods

### Tissue samples

A total of 25 human RCs and 25 human PGs were obtained for this study. The patients were randomly selected from those visiting the Endodontics department at the Faculty of Dentistry, Cairo University, for periapical surgery due to chronic periapical lesions that did not heal with routine endodontic therapy. Clinical and radiological examinations were conducted on the affected teeth. The study population included both genders (males and females), age ranging from 20 to 40 years, free from systemic disease, no pregnancy and receiving no medication as verified by a comprehensive medical history. Information concerning the patients’ demographics (gender and age) and their clinical characteristics (anatomical location, symptoms, and radiographic results) was gathered along with biopsy records. The control group comprised healthy dental pulp acquired from four individuals requiring surgical removal of sound third molars. These tissues exhibited no signs of inflammation and served as control specimens. Dental pulp, being a connective tissue with unique characteristics compared to other oral tissues, allows for a clear comparison of the inflammatory markers in periapical lesions without pre-existing inflammatory conditions [22].

Diagnosis of periapical lesions was made based on both histopathologic and clinical assessments. RCs were identified by the following criteria: (1) Existence of a lesion situated periapical to a non-vital tooth (2) Identification of a cavity containing fluid or semifluid during surgical exploration (3) Histological confirmation of nonkeratinizing stratified squamous epithelium either partially or entirely lining a cystic cavity or tissue samples. On the other hand, PGs consisted of granulomatous tissue containing numerous infiltrating inflammatory cells such as lymphocytes, macrophages, polymorphonuclear leukocytes, and plasma cells, without the presence of epithelial cells.

The biopsies of periapical lesions were excised and subsequently fixed in a 10% neutral formalin solution. They were then dehydrated using increasing concentrations of ethyl alcohol and cleared in xylene. After processing, the tissues were embedded in fresh paraffin wax and sectioned to a thickness of four microns. These sections underwent the following procedures:

### Histological examination

Hematoxylin and Eosin staining was carried out to examine the histological features of the specimens and to evaluate the degree of inflammatory infiltration.

### Morphological analysis

The severity of the inflammatory infiltrates was assessed using quantitative score-based method. Quantitative assessment of inflammatory cells was conducted in ten distinct areas of histological sections at × 400 magnification. The inflammatory reaction was graded based on the abundance and distribution of inflammatory cells within the field using a four-point scoring system: Score 1 revealed few or no inflammatory cells (lack of reaction), score 2 corresponded to fewer than 25 cells (mild reaction), score 3 represented 25–125 cells (moderate reaction), and score 4 corresponded to more than 125 cells (severe reaction). All scoring was conducted by a blinded examiner to maintain objectivity and reduce bias [23, 24].

### Immunohistochemical examination

After deparaffinization, the sections were cleaned in phosphate buffer saline (PBS) and submitted to antigen retrieval using citrate buffer, PH 6.0. To suppress endogenous peroxidases, the samples were subjected to hydrogen peroxide. Non-specific background staining was eliminated by incubation in bovine serum albumin. The sections were incubated with an anti-TGF-β (monoclonal mouse anti-human TGF-β, clone TB21, 1:1000 dilution, AbD Serotec, Albedo, Romania– code MCA797T) and anti-CD68 (monoclonal mouse anti-human CD68, clone KP1, 1:200 dilution, DAKO, Carpinteria, CA, USA– code M0814) primary antibodies at room temperature for an hour. Following a buffer wash, the sections were subsequently incubated with biotinylated secondary antibodies (DAKO– code K0492) for an additional 30 min, after which they were washed. Diaminobenzidine solution was utilized as a chromogen. Mayer’s hematoxylin was utilized as a counterstain and the negative controller in use was PBS.

### Morphometric analysis

Interpretation of the immuno-stained sections was performed using an image analyzer computer system with the software Leica Quin 500 (Leica Microsystem, Switzerland) to assess the area percentage of the immuno-staining. Tissue sections were analyzed using a light microscope (100× magnification) to recognize regions containing immunoreactive cells, and the chosen fields were then examined at 400× magnification. Areas with immunoreactive cells were analyzed in 10 indicative and successive microscopic fields in various sites. Statistical analysis of the obtained data from the computer image analyzer was done to compare the mean area percentage values between the control and experimental groups.

### Statistical analysis

The recorded data from the image analyzer underwent statistical analysis, and the results were summarized as means and standard deviations. Data was examined for normality, using the Kolmogorov–Smirnov test. The results indicated normally distributed (parametric) data; therefore, the ANOVA test was employed comparing the three groups for each marker. Subsequently, Post Hoc Tukey test was used for pairwise comparisons. The level of significance was established at *p* ≤ 0.05. Additionally, demographic and clinical aspects in the study population of PGs and RCs were assessed using the Fisher test, while age-related differences were performed using T- test. Non-parametric data in morphological analysis score were performed using Kruskal-Wallis-test. Pearson’s correlation coefficient was used to analyze correlations.

## Results

### Demographic data

An examination of all cases of PGs revealed an increased incidence in females (*n* = 19; 76%), mean age range 25.5 ± 5, patients who were asymptomatic (*n* = 17; 68%), had radiolucencies (*n* = 25; 100%), with a lesion size of < 1 cm (*n* = 21, 84%), and anatomic location in the anterior maxilla (*n* = 18; 72%). The 25 RCs were more common in males (*n* = 13; 52%), mean age range 32.5 ± 4.77. The individuals who had no symptoms (*n* = 20; 80%). Regarding the anatomical position and radiographic appearance, radiolucency was seen in all cases (100%) with a lesion size of ≥ 1 cm (*n* = 19, 76%), primarily in the maxilla’s anterior region (*n* = 16; 64%). The only significant distinctions were detected in mean age range and lesion size (*P* < 0.001) (Table 1).

**Table 1:** Demographic data and clinical presentation of the study population of PGs and RCs

Parameter	PGs			RCs			*p*-value
Total cases	25			25			
Gender	Male	N %	6 24%	Male	N %	13 52%	0.0792
	Female	N %	19 76%	Female	N %	12 44%	
Age	Mean ± SD	25.5 ± 5		Mean ± SD	32.5 ± 4.77		< 0.001*
Symptoms	Asymptomatic	N %	17 68%	Asymptomatic	N %	20 80%	0.52
	Symptomatic	N %	8 32%	Symptomatic	N %	5 20%	
Anatomic Location	Anterior maxilla	N %	18 72%	Anterior maxilla	N %	16 64%	0.762
	Other	N %	7 28%	Other	N %	9 36%	
Lesion size	≥ 1 cm	N %	4 16%	≥ 1 cm	N %	19 76%	< 0.001*
	< 1 cm	N %	21 84%	< 1 cm	N %	6 24%	

Clinical variables are presented as numbers and percentages. * Significant at p-value < 0.05

### Histopathological results

**Fig. 1:**
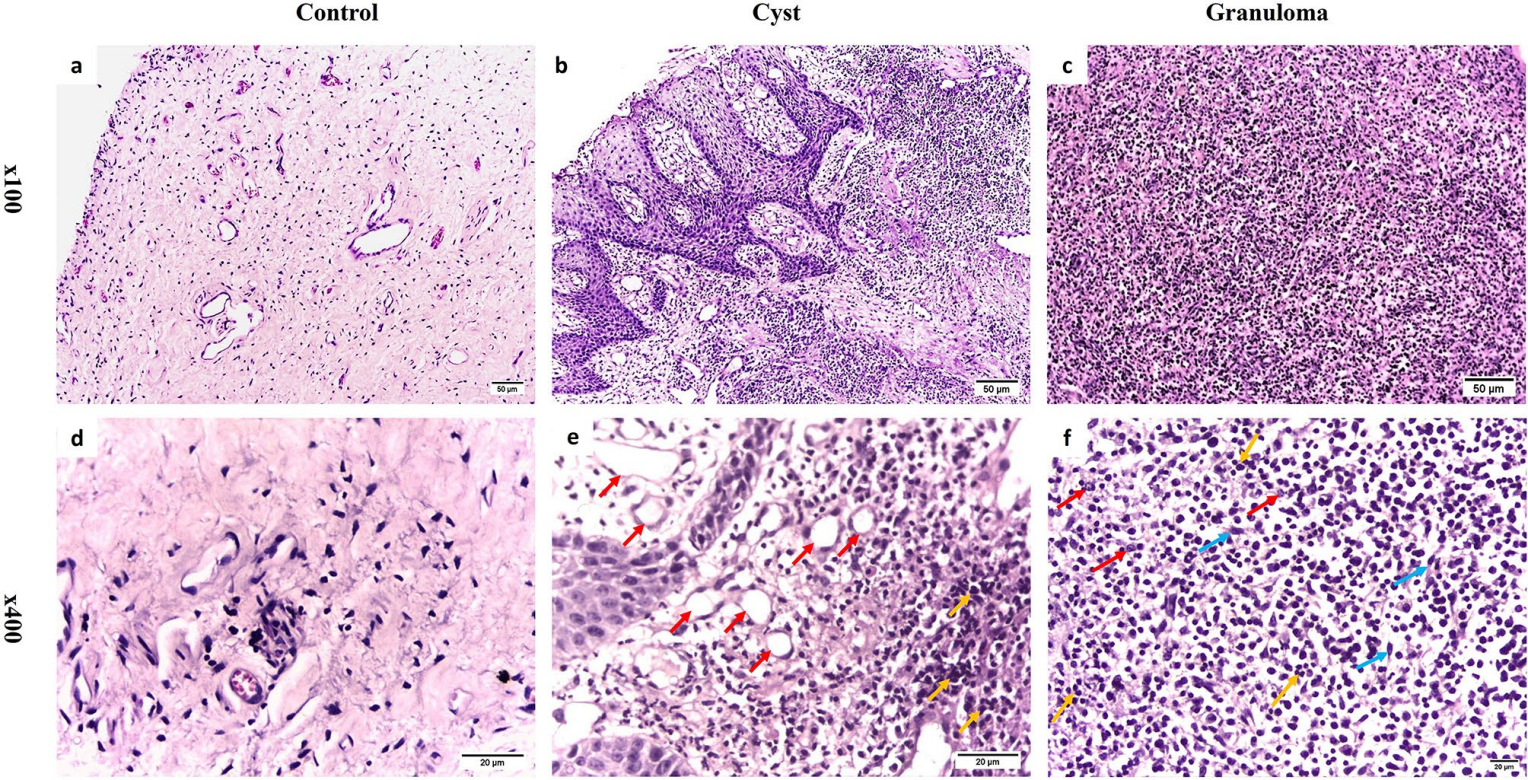
Representative histological photomicrographs using H&E staining. **a**, **d**. Healthy pulp tissues showing normal histology without any noticeable inflammatory cell infiltration. **b**, **e**. Radicular cyst revealing numerous inflammatory cell infiltration (orange arrows) & thin-walled dilated blood vessels (red arrows). **c**, **f**. Periapical granuloma displaying numerous inflammatory cell infiltration (orange arrows), fibroblasts (blue arrows) & plasma cells (red arrows) (**a**-**c** Orig. Mag. × 100; **d**-**f** Orig. Mag. × 400)

According to histopathology, the control group revealed normal and healthy pulp tissues. There was no evidence of inflammatory cell infiltration or capillary proliferation (Fig. 1a and d). On the other hand, the RCs appeared covered with a layer of nonkeratinized stratified squamous epithelium. This layer was connected to inflammatory fibrous connective tissue and a large infiltration of inflammatory cells. It displayed angiotelectasis along with extensive vascular hypertrophy and dilatation. Due to inflammatory hyperplasia, the luminal epithelium had a “looped and arcaded” appearance (Fig. 1b and e). Whereas the PGs were made up of granulation tissue that was inflamed and had intense inflammatory infiltrates. Macrophages, plasma cells, and lymphocytes made up most of the inflammatory cell infiltration (Fig. 1c and f).

### Morphological analysis results

Morphological analysis revealed the pulp tissues with no reaction with a median score of 1. However, distinct patterns of inflammatory response were observed between RCs and PGs. RCs showed moderate inflammatory reactions with a median score of 3, whereas PGs predominantly exhibited severe inflammatory reactions with a median score of 4. Statistical analysis done using Kruskal-Wallis-test showed a significant variation between groups (*p* < 0.001).

### Immunohistochemical results

**Fig. 2:**
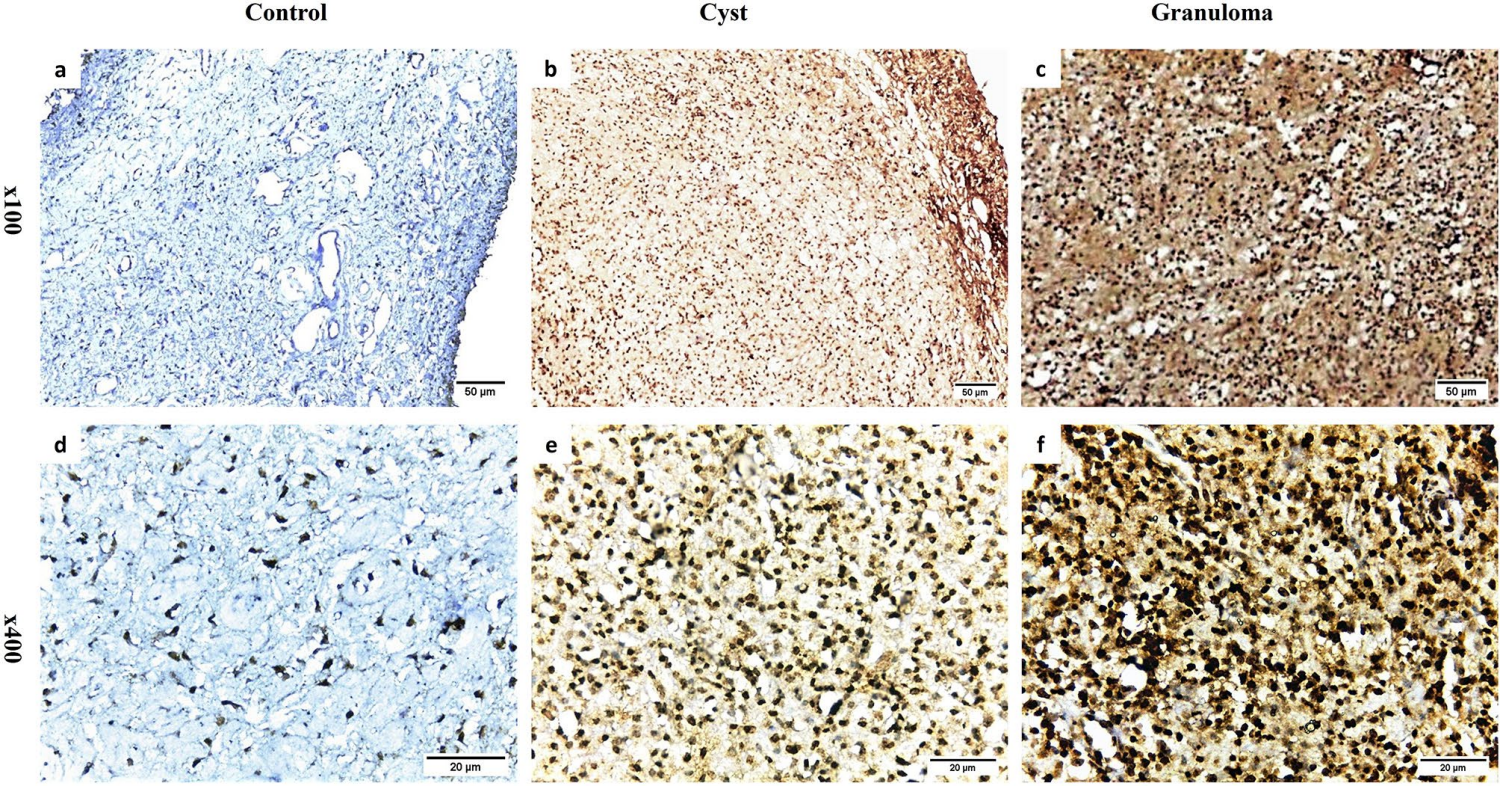
Representative photomicrographs of immunohistochemical staining of TGF-β1. **a**, **d**. weak staining in healthy pulp tissue; **b**, **e**. intense staining in radicular cyst; **c**, **f**. intense staining in periapical granuloma (**a**-**c** Orig. Mag. × 100; **d**-**f** Orig. Mag. × 400)

**Fig. 3:**
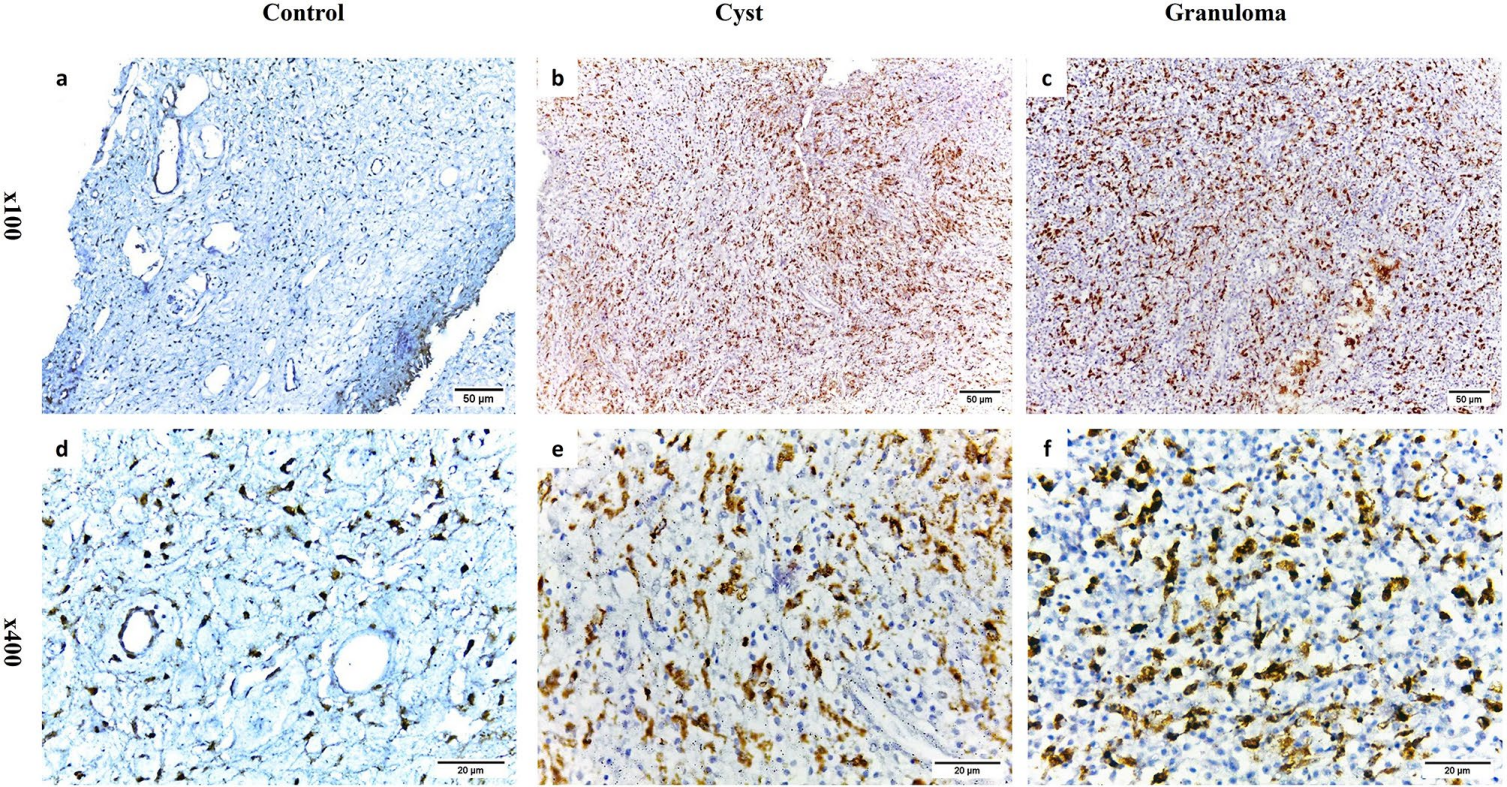
Representative photomicrographs of immunohistochemical staining of CD68 + cells. **a**, **d**. weak staining in healthy pulp tissue; **b**, **e**. intense staining in radicular cyst; **c**, **f**. intense staining in periapical granuloma (**a**-**c** Orig. Mag. × 100; **d**-**f** Orig. Mag. × 400)

Intensive staining for TGF-β1 and CD68 was detected in every sample of both RCs and PGs (Figs. 2b, e, c and f and 3b, e, c and f). Additionally, epithelial cells in RCs exhibited positive expression for TGF-β1 and CD68 (Figs. 2b and e and 3b and e). In contrast, healthy dental pulp tissues showed limited expression of TGF-β1 and CD68 (Figs. 2a and d and 3a and d).

### Morphometric analysis results

The area percentage of immunohistochemical staining in the lesions was quantified. Statistical analysis using ANOVA showed a significant disparity in all groups (*P* < 0.001). Multiple pairwise comparisons among groups demonstrated a significant elevation in both TGF-β1 and CD68 levels in RCs and PGs compared to the control group (normal pulp tissue) (*P* < 0.001). Furthermore, TGF-β1 and CD68 expression in PGs was significantly elevated than RCs (*P* < 0.001) (Table 2).

**Table 2:** Mean ± standard deviation of area percentage for TGF-β1 and CD68 among studied groups

Groups	Number	TGF-β1	CD68
		Mean ± SD	Mean ± SD
Control	4	0.142 ± 0.057 **c**	0.148 ± 0.066 **c**
RCs	25	25.43 ± 1.335 **b**	11.938 ± 1.136 **b**
PGs	25	52.75 ± 1.369 **a**	17.15 ± 1.016 **a**
P-value		< 0.001*	< 0.001*

* Significant difference between all groups using ANOVA at p-value < 0.05

Means sharing different letters in the same column are statistically significant from each other using post hoc Tukey’s test. All Post-HOC Tukey’s tests showed statistically significant difference (*P* < 0.001)

Pearson’s correlation among the examined markers in the granuloma and cyst groups revealed a negative correlation present among TGF-β1 and CD68 (*r* = -0.8803, *p* < 0.05; *r* = -0.1389, *p* < 0.05, respectively) (Table 3).

**Table 3:** Correlation between the immunoexpression of both studied markers in the granuloma and cyst groups

Variable		PGs		RCs	
		CD68	TGF-β1	CD68	TGF-β1
CD68	r p-value N	1.00 . 25	-0.8803 < 0.05* 25	1.00 . 25	-0.1389 < 0.05* 25
TGF-β1	r p-value N	-0.8803 < 0.05* 25	1.00 . 25	-0.1389 < 0.05* 25	1.00 . 25

r: correlation coefficient *Significant difference at p-value < 0.05

## Discussion

A histological analysis of periapical lesions is essential for evaluating clinical symptoms and radiographic signs, confirming periapical periodontitis diagnosis, and differentiating it from lesions that are not caused by inflammation [4].

In the herein study, healthy pulp tissue was chosen as a control due to challenges in obtaining periodontal ligament (PDL) tissues. Dental pulp tissues were deemed appropriate as they influence both inflammatory and healing processes, similar to PDL [22]. This methodological approach aligned with Zhu et al. [25]. Notably, several studies have excluded control groups entirely [26, 27]. While both approaches are valid, using pulp tissue as a control provides a practical and meaningful reference.

Histopathological examination of PGs and RCs in the current investigation revealed their typical histological pattern. Granulomas appeared as localized masses of chronic inflammatory tissue [28], whereas cysts were characterized by cystic formations with varying degrees of cell infiltration in their linings [29]. Additionally, both PGs and RCs exhibited severe inflammatory infiltration, consistent with previous findings emphasizing their inflammatory nature [30, 31].

The higher inflammatory cell infiltration observed in PGs compared to RCs can be attributed to the differing biological mechanisms underlying these lesions. Granulomas are active immune response to persistent stimuli, characterized by intense recruitment of macrophages, lymphocytes, and multinucleated giant cells. In contrast, RCs represent more stable lesions with localized inflammatory responses, primarily involving lymphocytes and plasma cells at the lesion’s periphery [32, 33].

TGF-β1 was investigated in this study due to its recognized role in both granulomas and cysts, suggesting its potential as a molecular marker for predicting periapical lesion healing [34]. The present work revealed significantly higher TGF-β1 expression in periapical lesions compared to normal pulp tissue, with PGs showing greater expression than RCs. This result corroborated findings by Álvares et al. [10], Marçal et al. [16], and Teixeira-Salum et al. [17].

Elevated TGF-β1 expression in PGs may reflect the unique immune and inflammatory microenvironments of these lesions. The persistent inflammatory activity in PGs likely drives increased TGF-β1 expression, contributing to immune regulation and tissue repair. Additionally, TGF-β1’s role in fibrosis and collagen deposition aligns with the granulomatous features of PGs [35, 36]. Conversely, the milder inflammatory activity in RCs may account for their relatively lower TGF-β1 expression [10, 37].

Macrophages serve as the predominant immune cells found in periapical lesions, infiltrating the tissue during its early stages [38]. In this work, the identification of CD68, a widely used macrophage marker, highlighted the essential role of macrophages in the pathophysiology of PGs and RCs.

In the herein study, CD68 was notably elevated in both PGs and RCs compared to normal pulp tissue, consistent with prior research [16, 39, 40]. Furthermore, previous research reported high CD68 + cell density in regions of active inflammation, such as the center of PGs and subepithelial zones of RCs [18, 41].

The higher number of CD68 + macrophages in PGs compared to RCs aligned with de Farias et al. [27], who reported similar findings. This increase may reflect macrophages’ critical roles in phagocytosis, immune activation, antigen presentation, and tissue remodeling—all processes characteristic of granulomas [42, 43].

However, some studies reported no significant differences in CD68 + cell expression between PGs and RCs [39, 44, 45], while Bracks et al. [41] documented higher macrophage counts in RCs. These discrepancies could stem from variations in experimental design, including antibody selection, staining protocols, and tissue preparation techniques.

Demographic data from this study were consistent with previous epidemiological research [10, 46–48]. Notably, smaller PGs exhibited higher marker expression levels compared to larger RCs, a finding supported by França et al. [31] who reported higher CD68 immunoexpression in smaller cysts and lower expression in larger cysts. Similarly, since TGF-β1 is secreted by macrophages, this might explain its higher expression in PGs regardless of lesion size. However, some studies have linked TGF-β1 levels to lesion size, attributing increased expression to heightened cell activation or density in larger lesions [34].

Most lesions in the current work were asymptomatic but exhibited varying levels of marker expression. This aligned with previous research [10, 14, 49] showing elevated TGF-β1 and other immunoregulatory cytokines in asymptomatic lesions, suggesting a role of these cytokines in tissue homeostasis and inflammation modulation. Similarly, Azeredo et al. [45] reported no significant differences in macrophage percentages between symptomatic and asymptomatic lesions.

In terms of clinical significance, these findings emphasize that periapical lesions often exhibit overlapping clinical features, complicating accurate diagnosis. Therefore, clinicians should prioritize histopathological evaluation to ensure reliable diagnosis and informed treatment planning.

In the current analysis, TGF-β1 and CD68 showed a negative correlation in both PGs and RCs, despite theoretical associations reported in the literature [50]. A plausible explanation is TGF-β1’s dual role in immune regulation [12, 13]. Initially, it promotes macrophage recruitment during early inflammation. As the inflammatory response progresses, TGF-β1 exerts immunosuppressive effects, resulting in a reduction in macrophage activity and infiltration. This dynamic shift from proinflammatory to anti-inflammatory functions may account for the observed negative correlation [51].

### Limitations

This study has some limitations that should be carefully considered when interpreting the results. The relatively small sample size may limit the generalizability of the findings, highlighting the need for studies with larger cohorts to validate these results. Additionally, the reliance on soft tissue biopsies restricts the scope of this study to soft tissue involvement, thereby limiting insights into hard tissue changes such as bone resorption or remodeling. The cross-sectional design further constrains the ability to evaluate the temporal progression of TGF-β1 and CD68 expression across different stages of lesion development and healing. Furthermore, the use of normal pulp tissue as a control, due to the unavailability of PDL tissues, may impact the contextual interpretation of findings related to periapical pathophysiology. Addressing these limitations in future longitudinal and comparative studies will provide deeper insights into the role of these markers in periapical lesions.

## Conclusion

This study confirmed the critical role of macrophages and TGF-β1 in the pathogenesis of human periapical lesions, with distinct expression patterns observed in RCs and PGs. These markers reflect the inflammatory activity and tissue remodeling processes that are characteristic of these lesions. While our findings highlighted differences in TGF-β1 and CD68 expression, their precise roles in periapical tissue repair and resolution remain uncertain. This underscores the necessity for further research to clarify their mechanistic contributions. Future studies should focus on exploring the temporal dynamics of these markers across various stages of lesion development and healing to determine their potential as therapeutic targets. Such investigations could pave the way for innovative strategies to improve clinical outcomes in endodontic treatments, particularly in managing chronic periapical conditions.

## Data Availability

All data generated or analyzed during this study are included in this published article.

## Abbreviations

AP: Apical Periodontitis
TGF-β1: Transforming Growth Factor Beta 1
CD68: Cluster of Differentiation 68
RCs: Radicular Cysts
PGs: Periapical Granulomas
PBS: Phosphate Buffer Saline
